# Polydactyly 24 in a Female Neonate

**DOI:** 10.1155/2013/798138

**Published:** 2013-09-05

**Authors:** Oluseyi O. A. Atanda, Kola M. Owonikoko, Adewale S. Adeyemi, Olanrewaju Bajowa

**Affiliations:** ^1^Department of Obstetrics and Gynaecology, Ladoke Akintola University of Technology (LAUTECH) Teaching Hospital, PMB 4000, Oyo State, Ogbomoso, Nigeria; ^2^Department of Obstetrics and Gynaecology, Faculty of Clinical Sciences, College of Health Sciences, Ladoke Akintola University of Technology (LAUTECH), PMB 4007, Oyo State, Ogbomoso, Nigeria

## Abstract

Polydactyly is perhaps one of the most common congenital hand and foot anomalies. Tetrapolydactyly (polydactyly 24) is a very rare form of hand and foot anomalies. Postaxial ray polydactyly usually occurs in male blacks without associated congenital abnormalities. We report a case of postaxial ray tetrapolydactyly in a female neonate which occurred sporadically and without associated congenital abnormalities.

## 1. Introduction

Polydactyly is one of the most common congenital anomalies of the hands and feet consisting of supernumerary fingers or toes [1, 2]. The extra digit is usually a small piece of soft tissue. Occasionally, it may contain bone without joints; it may be a complete functioning digit [2]. This condition can occur in one limb or can be exceptionally present in all four limbs a condition called tetrapolydactyly [3].

The extra digit is the most common on the ulnar side of the hand—post axial ray, less common on the radial side—preaxial ray, and very rarely within the middle three digits—middle or central ray [2, 3]. It can occur as an isolated disorder, in association with other malformations of the hands or feet, or as part of a syndrome. These syndromes include Holt-Oram syndrome, Down's syndrome, Fanconi Polycythemia, Meckel syndrome, Laurence-Moon-Biedl syndrome, Patau's syndrome, and Klippel-Trenaunay syndrome. There may be associated cleft palate, hearing difficulties, renal anomalies, and other limb and vertebral anomalies [4].

It can occur sporadically, but it can also be inherited with a mainly autosomal dominant inheritance [1]. Polydactyly commonly involves only the hand or the foot.

Postaxial hand polydactyly is a common isolated disorder in African black and African American children, and autosomal dominant transmission is suspected. Postaxial polydactyly is more frequent in blacks than in whites and is more frequent in male children. In contrast, postaxial polydactyly seen in white children is usually syndromic and associated with an autosomal recessive transmission [3].

 Polydactyly involving both hands and feet is rare [5]. We report a case of tetrapolydactyly (polydactyly 24) in a female neonate.

## 2. Case Presentation

Baby A.T. is a female neonate who was delivered through spontaneous vaginal delivery to a Para 5+1 (5 alive) trader. Baby was delivered at 39 weeks and weighed 3600 grammes with head circumference and body length within normal range.

There was no family history for hand and foot malformations in her other children or in her own family or that of her husband. There was no history of drug ingestion other than the routine drugs prescribed to her during antenatal care.

Examination of the newborn revealed hand and foot polydactyly with 6 fingers bilaterally and 6 toes at both feet. Other than these findings, there were no other malformations or conditions noted.

Further examination of the hands and feet did not reveal involvement of bones other than soft tissue which was confirmed by X-ray. Clinico-radiological examination revealed no other congenital anomaly. The child was followed up to 6 months without any other abnormality identified.

## 3. Discussion

Polydactyly is perhaps the most common congenital hand anomaly [2]. Various incidences have been reported but on average the incidence in blacks is about 1 in 300 while in white it is about 1 in 3000 [6]. Incidence of tetrapolydactyly was not found as it has been reported to be very rare [5]. It has also been said to be commoner in males and most cases of polydactyly reported in literature conforms to this. In this case presentation, the patient is a female of Nigerian parents with no family history of such malformations. This patient was delivered in our hospital which is a new teaching hospital which was established in 2010 and has had a total of 1847 deliveries in two years. This gives an incidence of 1 in 1800. 

Post axial ray polydactyly has been reported to be the common presentation in blacks, usually in males and an isolated disorder [2]. The occurrence of either polydactyly or tetrapolydactyly in females has been rarely reported. Radulescu et al. had reported a case of a female neonate with tetrapolydactyly in a family where 3 generations of the males were affected by polydactyly or just isolated hand or foot malformations [7]. This is also a case of isolated post axial ray tetrapolydactyly in a female neonate without any other congenital anomaly and no similar occurrence in her male siblings or parents. Polydactyly in blacks usually occurs in isolation.

**Figure 1:**
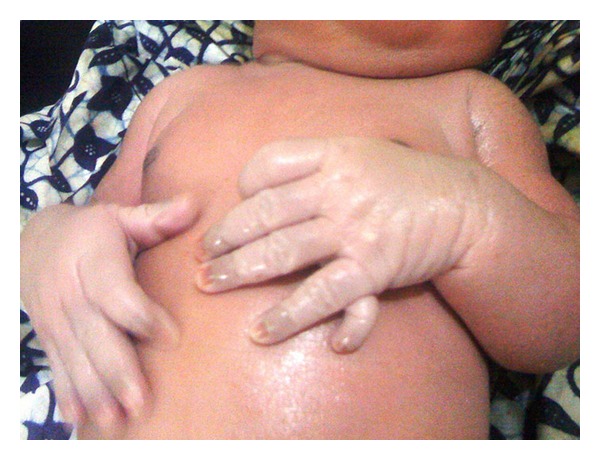
The 6 digits of left hand. The cutaneous nubbin is seen on the ulnar side of both hands.

**Figure 2:**
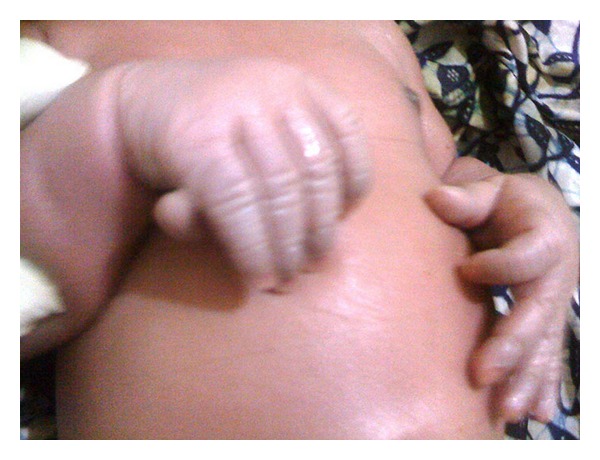
The 6 digits of right hand. The cutaneous nubbin is seen on the ulnar side of both hands.

**Figure 3:**
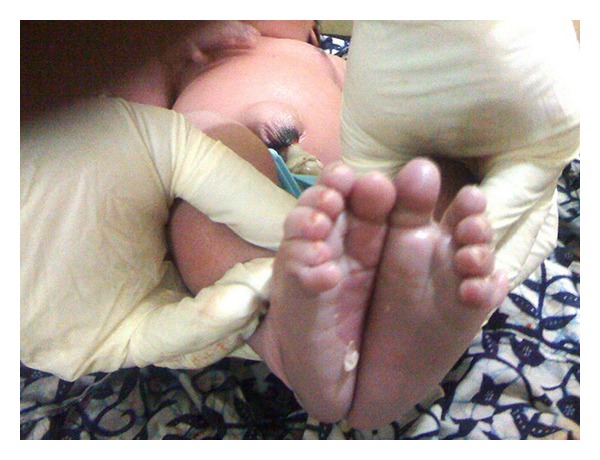
Shows both feet with the extra digits.

In polydactyly, extra digit may be functional or nonfunctional. Extra digit usually lacks muscular connections [8]. In this case, we report a non-functional digit in both hands and feet with no tendon or muscle attached over the extra digits. They are fleshy nubbins also reported as a form of classification [9]. The majority of cases of polydactyly without bony involvement usually have the extra digits tied off at birth [6]; this was also done for this female neonate at birth (Figures 1–3).

In conclusion, tetrapolydactyly still remains a rare occurrence globally, and its occurrence in a female, though without associated congenital anomalys is worth reporting.
